# Using Simulation to Compare Established and Emerging Interventions to Reduce Cardiovascular Disease Risk in the United States

**DOI:** 10.5888/pcd11.140130

**Published:** 2014-11-06

**Authors:** Jack Homer, Kristina Wile, Benjamin Yarnoff, Justin G. Trogdon, Gary Hirsch, Lawton Cooper, Robin Soler, Diane Orenstein

**Affiliations:** Author Affiliations: Jack Homer, Homer Consulting, Barrytown, New York; Kristina Wile, Sustainability Institute, Charleston, South Carolina; Justin G. Trogdon, University of North Carolina, Chapel Hill, North Carolina; Gary Hirsch, Creator Learning Environments, Wayland, Massachusetts; Lawton Cooper, National Institutes of Health, Bethesda, Maryland; Robin Soler, Diane Orenstein, Centers for Disease Control and Prevention, Atlanta, Georgia.

## Abstract

**Introduction:**

Computer simulation offers the ability to compare diverse interventions for reducing cardiovascular disease risks in a controlled and systematic way that cannot be done in the real world.

**Methods:**

We used the Prevention Impacts Simulation Model (PRISM) to analyze the effect of 50 intervention levers, grouped into 6 (2 x 3) clusters on the basis of whether they were established or emerging and whether they acted in the policy domains of care (clinical, mental health, and behavioral services), air (smoking, secondhand smoke, and air pollution), or lifestyle (nutrition and physical activity). Uncertainty ranges were established through probabilistic sensitivity analysis.

**Results:**

Results indicate that by 2040, all 6 intervention clusters combined could result in cumulative reductions of 49% to 54% in the cardiovascular risk-related death rate and of 13% to 21% in risk factor-attributable costs. A majority of the death reduction would come from Established interventions, but Emerging interventions would also contribute strongly. A slim majority of the cost reduction would come from Emerging interventions.

**Conclusion:**

PRISM allows public health officials to examine the potential influence of different types of interventions — both established and emerging — for reducing cardiovascular risks. Our modeling suggests that established interventions could still contribute much to reducing deaths and costs, especially through greater use of well-known approaches to preventive and acute clinical care, whereas emerging interventions have the potential to contribute significantly, especially through certain types of preventive care and improved nutrition.

## Introduction

Heart disease and stroke are the first and fourth leading causes of death in the United States (1) despite being largely preventable. This situation has raised the call for public health intervention to improve prevention. Policy makers have a range of choices for public health intervention to increase prevention through modifiable risks, such as smoking, obesity, stress, air pollution, poor diet, and physical inactivity. The importance of intervening to limit these risks is described in *A Public Health Action Plan to Prevent Heart Disease and Stroke* (2) and highlighted by the Million Hearts initiative (http://millionhearts.hhs.gov/index.html).

Risk factors can be addressed by implementing established interventions with a scientific evidence base and emerging interventions with a growing evidence base. To classify interventions as either emerging or established, we conducted a review of guidance from the *Guide to Community Preventive Services* (3), Cochrane Reviews (4), Health Evidence Reviews (5), US Preventive Services Task Force (6), National Institutes of Health (7), American Heart Association (8), American College of Physicians (9), US Department of Agriculture (10), Centers for Disease Control and Prevention (11), American Psychiatric Association (12), American Diabetes Association (13), and American Academy of Sleep Medicine (14). If more than 50% of the published guidance documents relating to an intervention from these sources found sufficient evidence to recommend it, we classified the intervention as established. If less than 50% of the published guidance documents recommended the intervention or if there was no guidance related to the intervention, we classified it as emerging.

Computer simulation permits comparison of diverse interventions in a controlled and systematic way that cannot be done in the real world. Simulating various interventions individually and in combination and testing across their ranges of uncertainty can help policy makers evaluate how to balance investments in established and emerging interventions. In this analysis, we used the Prevention Impacts Simulation Model (PRISM) to compare established and emerging prevention interventions across the 3 domains of care, air, and lifestyle, in terms of their potential for reducing the adverse consequences of cardiovascular disease (CVD) risk factors in the US population through the year 2040. This analysis can help inform the policy conversation about what kinds of prevention and care-based interventions are most useful for addressing CVD (2,15–17).

## Methods

### PRISM and its intervention levers

PRISM is a nationally representative computer model that simulates the effects of diverse clinical and population-level interventions aimed at reducing risks for CVD events (ie, acute events coded as coronary heart disease, stroke, heart failure, or peripheral artery disease) and for other chronic conditions and diseases (eg, hypertension, diabetes, renal disease, obstructive pulmonary disease, certain cancers) (15,18,19). It is a compartmental system dynamics model and, like other such models, depicts processes of multiple and nonlinear influence, accumulation, delay, and feedback that result in movements among healthy and ill population subgroups (20–22). The conceptual and numerical assumptions of PRISM are drawn from scientific literature, national survey data, and subject matter expertise. PRISM provides a user-friendly interface and in seconds produces 50 years of simulated output, from 1990 through 2040, answering “what if” questions posed in interactive workshops and study sessions. The model has been calibrated to represent the United States and some local areas (18,23,24).

In PRISM, the population is segmented by 6 childhood and adult age categories, by sex, and by CVD event status (not-yet or non-CVD, and already-had or post-CVD). The model simulates changes in the population through birth, death, migration, aging, and movement from non-CVD to post-CVD status. It also depicts flows into and out of 3 blood pressure categories (normal, borderline, high), 3 blood cholesterol categories (normal, borderline, high), 3 blood glucose categories (normal, prediabetic, diabetic), 4 smoking categories (never, current, recent ex-smoker, long-term ex-smoker), and 2 body mass index categories (non-obese, obese). PRISM also includes risk factors of poor nutrition (ie, inadequate consumption of fruits and vegetables and excess consumption of junk food, sodium, or trans fats), inactivity, psychological distress, secondhand smoke, periodontal disease, sleep apnea, small particulate air pollution, and inadequate use of aspirin for primary prevention. Co-occurrence of these risk factors in individuals is not modeled explicitly (PRISM is a compartmental model of subpopulations rather than separate individuals), but interactions among the risk factors are represented algebraically in equations for cardiovascular events and deaths and also in equations describing how the prevalence of one risk factor (eg, smoking) may affect another (eg, diabetes). The model simulates changes by quarter-year increments in more than 4,000 variables, including changes in population mix, risk factor prevalence, and risk factor–related morbidity, mortality, and costs (19).

The current version of PRISM, version 3p, includes 50 intervention levers that may prevent or mitigate the cardiovascular risks described above. All interventions in the model are based on peer-reviewed literature and discussions with experts working in the field who helped to specify and quantify their potential effects (19).

### Testing 6 intervention clusters

We grouped PRISM’s 50 intervention levers into established and emerging interventions that we crossed with policy categories of care (clinical, behavioral, and mental health services), air (smoking, secondhand smoke, and air pollution), and lifestyle (nutrition and physical activity), resulting in 6 clusters (Table 1). For each intervention, the table gives several pieces of information: estimated size of the target population in 2010, the existing recipient population in 2010 (for care interventions), the unit cost per recipient per year (for care interventions), the initial (pre-2012) lever setting for the intervention, and the best plausible (full extent) lever setting (19).

**Table 1:** Interventions, Target Populations, and Risk Factor Management Costs in the Prevention Impacts Simulation Model (PRISM)

PRISM Intervention Lever	Estimated Target Population 2010, millions	Recipient Population 2010, if Applicable, millions	Unit Cost per Recipient per Year, if Applicable, 2008 $	Initial Lever Setting	Best Plausible Lever Setting^a^
**Care established: 22 intervention levers**					
Use of quality BP care: non-CVD, post-CVD	78.7^b^	39.4	440	60	100
Use of quality cholesterol care: non-CVD, post-CVD	108.6^c^	35.7	420	55	100
Use of quality diabetes care: non-CVD, post-CVD	26.4^d^	11.2	1,700	57	100
Use of quality acute and rehabilitative care	3.7^e^	3.0	26,050	80	100
Use of quality CVD care post-CVD	27.4^f^	19.2	2,000	70	100
Use of quit counseling and NRT by smokers	51.1^g^	5.1	619	10	20
Use of weight loss services by obese	75.3^h^	7.5	650	10	24
Fraction of diagnosed sleep apnea	34.3^i^	2.9	600	33	100
Fraction of individuals with sleep apnea that own a CPAP	34.3^i^	2.9	600	65	100
Fraction of individuals that own a CPAP who use it	34.3^i^	2.9	600	40	100
Fraction of individuals that use a CPAP who use it effectively	34.3^i^	i2.9	600	75	100
Regular dental care to prevent periodontal disease: non-CVD, post-CVD	129.7^j^	55.8	300	43	100
Aspirin use by eligible non-CVD population: male, aged <65 y	39.5^k^	11.8	14	30	100
Aspirin use by eligible non-CVD population: female, aged <65 y	18.8^k^	7.1	14	38	100
Aspirin use by eligible non-CVD population: male, aged ≥65 y	13.1^k^	7.9	14	60	100
Aspirin use by eligible non-CVD population: female, aged ≥65 y	15.6^k^	8.9	14	57	100
Use of support services by distressed: non-CVD	28^l^	5.7	2,080	20.6	54
Use of support services by distressed: post-CVD	6.9^l^	1.6	2,080	23.4	80
**Care emerging: 12 intervention levers**					
Borderline BP care: non-CVD, post-CVD	17.4^m^	0	220	0	100
Tighter BP care: non-CVD, post-CVD	78.7^n^	0	88	0	100
Borderline cholesterol care: non-CVD, post-CVD	35.8^o^	0	378	0	100
Tighter cholesterol care: non-CVD, post-CVD	108.6^p^	0	126	0	100
Prediabetes care: non-CVD, post-CVD	73.3^q^	0	850	0	100
Tighter diabetes care: non-CVD, post-CVD	26.4^r^	0	850	0	100
**Air established: 3 intervention levers**					
Tobacco tax rate	253.4^s^	—	—	34	100
Tobacco marketing restriction index	253.4^s^	—	—	25	100
Fraction of workplaces allowing smoking	253.4^s^	—	—	11	0
**Air emerging: 2 intervention levers**					
Tobacco counter-marketing index	253.4^s^	—	—	20	100
Average small particulate air pollution (µg/cubic meter PM 2.5)	295.4^t^	—	—	10.9	7.0
**Lifestyle established: 3 intervention levers**					
Physical activity facilities access index	295.4^t^	—	—	61	100
Physical activity promotion index	295.4^t^	—	—	1	100
Physical activity in schools index	50.1^u^	—	—	27	100
**Lifestyle emerging: 8 intervention levers**					
Average sodium consumption (mg/d): hypertensives	78.7	—	—	3,700	1,850
Average sodium consumption (mg/d): nonhypertensives	216.8	—	—	4,000	2,000
Trans fat fraction of calories	295.4^t^	—	—	1.3	0
Junk food tax rate	295.4^t^	—	—	1	20
Junk food counter-marketing index	295.4^t^	—	—	0	100
Fruit and vegetable access index	295.4^t^	—	—	75	100
Fruit and vegetable promotion index	295.4^t^	—	—	2.5	100
Physical activity in childcare index	16.8^v^	—	—	30	100

Abbreviations: BP, blood pressure; CVD, cardiovascular disease; post-CVD, already diagnosed with CVD; non-CVD, not yet diagnosed with CVD; NRT, nicotine replacement therapy; CPAP, continuous positive airway pressure; PM 2.5, particles smaller than 2.5 µm,—, not applicable.

a Values are percentages unless otherwise indicated.

b Ever told they had high blood pressure or systolic blood pressure (SBP) at or greater than 140 mm Hg or diastolic blood pressure (DBP) at or greater than 90 mm Hg.

c Ever told they had high cholesterol or low-density lipoprotein (LDL) cholesterol at or greater than 130 mg/dL.

d Ever told they had diabetes or fasting glucose at or greater than 126 mg/dL.

e Fraction of cardiovascular event patients arriving timely to hospital, receiving recommended in-hospital care, and receiving rehabilitative care as appropriate.

f Fraction of post-CVD patients receiving preventive medications or elective revascularization as appropriate.

g Smokers who have smoked within past 6 months and had at least 100 cigarettes in their lifetime.

h Youth obesity defined as greater than 95% percentile by sex and age on Centers for Disease Control and Prevention growth chart from 1970s. Adult obesity defined as body mass index at or greater than 30 kg/m^2^.

i Ever told they had obstructive sleep apnea or sleep-disordered breathing.

j Population of individuals with gingivitis or periodontal disease. Periodontal disease is defined as at least 1 site with at or greater than 3 mm clinical attachment loss and at or greater than 4 mm pocket depth.

k Use every day or every other day, as a fraction of those eligible per the US Preventive Services Task Force (27): men aged 45 to 79, women aged 55 to 79.

l Score at or greater than 6 on Kessler-6 questionnaire for serious psychological distress (28).

m Borderline high blood pressure defined as SBP at or greater than 130 mm Hg or DBP at or greater than 85 mm Hg but not high blood pressure by definition above.

n Tight control of high blood pressure achieves SBP less than 130 mm Hg and DBP less than 85 mm Hg.

o Borderline high cholesterol defined as LDL cholesterol at or above 110 mg/dL but not high cholesterol by definition above.

p Tight control of high cholesterol achieves LDL cholesterol lower than 100 mg/dL.

q Prediabetes defined as fasting glucose at or greater than 100 mg/dL but not diabetes by definition above.

r Tight control of diabetes achieves HbA1c lower than 5.7%.

s Total population aged 12 or older.

t Total population aged 2 or older.

u Total population aged 6 through 11.

v Total population aged 2 through 5.

Within the care policy domain, the established interventions include the use of quality preventive care for diagnosis and basic control (that is, according to current standards) of high blood pressure, high cholesterol, and diabetes; quality acute and rehabilitative care after cardiovascular events; quality care for post-CVD patients to prevent recurrent events; behavioral services for smoking cessation and weight loss for the obese; mental health services to address psychological distress and depression; diagnosis of sleep apnea and its effective treatment through the use of continuous positive airway pressure (CPAP) devices; prevention of periodontal disease through regular dental care; and the use of aspirin for primary prevention among middle-aged and older people (men aged 45 to 79, women aged 55 to 79) who have not had a cardiovascular event. Emerging care interventions include tighter control (that is, based on lower thresholds than current standards) of high blood pressure, high cholesterol, and diabetes and the control of borderline hypertension, borderline cholesterol, and prediabetes.

Within the air domain, the established interventions include several policies for tobacco: taxation, counter-marketing, and prohibitions on workplace smoking. Emerging interventions in the air domain are tobacco marketing restrictions and the reduction of small particulate (PM 2.5, also known as soot) air pollution. Although the sources of PM 2.5 are well understood and reductions of 50% or more were achieved from 1990 to 2010, no consensus exists on how to accomplish further reduction as a matter of national policy (25).

Within the lifestyle domain, the established interventions consist of policies for increasing access to and promoting physical activity for adults and older children. Emerging interventions in this domain include strategies to reduce sodium and trans fat consumption (through legislative or market-based actions), the taxation and counter-marketing of energy-dense (junk) food, improving physical activity in child care, and policies for increasing access to and promoting the consumption of fruits and vegetables.

In this analysis, we report the results of testing these 6 clusters one at a time and in combination. When an intervention lever was included in a cluster being tested, it was ramped up linearly to a level of best plausible implementation from 2012 to 2017 and remained in full effect thereafter to the end of the simulation in 2040. For some of the interventions (whether established or emerging), the best plausible lever setting was directly measurable (eg, milligrams of sodium consumed daily, fraction of workplaces allowing smoking), whereas for others it was defined as 100% of an ideal or best practice as described in the literature or by subject matter experts.

We report annual outcome variables that include preventable deaths per thousand adults (death rate) that include CVD deaths and non-CVD deaths attributable to CVD risk factors, such as chronic obstructive pulmonary disease, end-stage renal disease, and various cancers (lung, colorectal, breast, and others); years of potential life lost per thousand adults; 3 categories of per-adult cost (direct risk factor management costs, direct acute and extended care costs, and indirect productivity costs); and the sum of these per-adult costs (19). Costs were discounted by 3% per year back to 2012. Cumulative effects are reported as of 2020 and as of 2040, in terms of percentage changes relative to the status quo base case of no change in intervention levers after 2012.

To test the robustness of results, each of the 6 cluster intervention scenarios was tested by using probabilistic sensitivity analysis. In 200 Monte Carlo runs of PRISM, 89 different parameters quantifying intervention effect sizes were set to randomly selected values along assumed uniform probability distributions with estimated mean, minimum, and maximum. These distributional parameters were taken from means and confidence intervals reported in the research literature where available and otherwise from the estimates of subject matter experts (19).

## Results

### Cumulative results through 2020

We calculated cumulative results from testing the 6 intervention clusters (care, air, and lifestyle in both established and emerging interventions) from 2012 to 2020 (Table 2). Of the 3 policy domains, combining the care interventions led to the greatest reduction in the death rate (−30.4%; 95% uncertainty range, −33.5% to −29.3%) and per capita acute and extended care costs (−17.9%; 95% uncertainty range, −21.6% to −15.6%). However, the care interventions also led to a substantial increase in risk factor management costs (105.8%; 95% uncertainty range, 94.9% to 117.4%), which includes the direct costs of office visits, medications, equipment, and ambulatory medical procedures. As a result, the care interventions together did not lead to much, if any, reduction in combined costs (−0.8%; 95% uncertainty range, −4.4% to 1.7%). The health effects were greater for the established care interventions than for the emerging care interventions, but both made significant contributions.

**Table 2:** Grouped Intervention Impacts on Deaths and Costs, 2012 Through 2020

Outcome	% Change from Base Case^a^ (95% Uncertainty Range)^b^			Base Case^a^
	Established	Emerging	Combined	
**Care interventions^c^**				
Death rate^d^	−23.3 (−26.4 to −20.4)	−9.4 (−9.7 to −9.2)	−30.4 (−33.5 to −29.3)	5.17
YPLL rate^e^	−22.3 (−25.1 to −19.7)	−8.2 (−8.5 to −7.9)	−28.4 (−31.1 to −27.5)	66.7
Risk management costs^f^	66.6 (61.6 to 71.0)	24.4 (19.5 to 30.6)	105.8 (94.9 to 117.4)	612
Acute and extended care costs^g^	−11.7 (−15.3 to −8.1)	−5.4 (−5.8 to −5.0)	−17.9 (−21.6 to −15.6)	636
Productivity costs^h^	−19.7 (−22.4 to −17.2)	−7.2 (−7.4 to −7.0)	−25.3 (−27.6 to −23.8)	2,217
Combined costs^i^	−3.0 (−5.7 to −0.2)	−1.3 (−2.2 to −0.2)	−0.8 (−4.4 to 1.7)	3,466
**Air interventions^c^**				
Death rate^d^	−0.9 (−1.1 to −0.7)	−1.84 (−1.8 to −1.2)	−2.3 (−2.6 to −1.9)	5.17
YPLL rate^e^	−0.8 (−1.0 to −0.6)	−1.2 (−1.5 to −1.0)	−2.0 (−2.3 to −1.7)	66.7
Risk management costs^f^	−0.4 (−0.6 to −0.3)	−0.1 (−0.2 to −0.1)	−0.6 (−0.7 to −0.4)	612
Acute and extended care costs^g^	−0.8 (−1.0 to −0.6)	−1.4 (−1.7 to −1.1)	−2.1 (−2.5 to −1.8)	636
Productivity costs^h^	−1.1 (−1.4 to −0.9)	−1.1 (−1.4 to −0.9)	−2.2 (−2.5 to −1.9)	2,217
Combined costs^i^	−1.0 (−1.2 to −0.7)	−1.0 (−1.2 to −0.8)	−1.9 (−2.2 to −1.6)	3,466
**Lifestyle interventions^c^**				
Death rate^d^	−0.4 (−0.6 to −0.1)	−5.5 (−7.0 to −4.0)	−5.7 (−7.3 to −4.4)	5.17
YPLL rate^e^	−0.4 (−0.7 to −0.2)	−4.7 (−6.1 to −3.5)	−5.1 (−6.5 to −3.9)	66.7
Risk management costs^f^	−0.3 (−0.5 to −0.1)	−3.5 (−4.2 to −2.7)	−3.8 (−4.5 to −2.9)	612
Acute and extended care costs^g^	−0.3 (−0.4 to −0.1)	−4.2 (−5.7 to −2.8)	−4.4 (−6.0 to −3.1)	636
Productivity costs^h^	−0.4 (−0.7 to −0.2)	−4.1 (−5.2 to −3.0)	−4.4 (−5.6 to −3.4)	2,217
Combined costs^i^	−0.4 (−0.6 to −0.1)	−4.0 (−5.1 to −3.1)	−4.3 (−5.5 to −3.3)	3,466
**All 3 intervention areas^c^**				
Death rate^d^	−23.8 (−26.9 to −20.9)	−14.7 (−15.9 to −13.6)	−32.6 (−34.7 to −30.6)	5.17
YPLL rate^e^	−22.8 (−25.5 to −20.3)	−12.8 (−13.8 to −11.8)	−30.4 (−32.2 to −28.7)	66.7
Risk management costs^f^	65.1 (60.4 to 69.5)	20.0 (14.9 to 26.0)	95.0 (84.8 to 106.7)	612
Acute and extended care costs^g^	−12.4 (−15.9 −8.9)	−10.1 (−11.7 to −8.6)	−21.2 (−24.1 to −18.1)	636
Productivity costs^h^	−20.5 (−23.1 to −18.0)	−11.1 (−11.9 to −10.3)	−27.2 (−29.0 to −25.4)	2,217
Combined costs^i^	−3.9 (−6.6 to −1.1)	−5.8 (−6.7 to −4.1)	−4.5 (−7.4 to −1.7)	3,466

Abbreviation: YPLL, years of potential life lost.

a Base case results are cumulative averages from model simulations with only baseline conditions for 2012 through 2020.

b Percentage changes from base case with uncertainty ranges are comparisons of intervention test results against base case for 200 different model Monte Carlo calibrations defined by random sampling of 89 uncertain effect-size parameters. The number not in parentheses is the median of the 200 comparisons; the 2 numbers in parentheses are the 2.5 percentile and 97.5 percentile of the 200 comparisons, respectively.

c Interventions ramp up to full strength over 5 years, from 2012 to 2017.

d Death rate refers to deaths per thousand adults from cardiovascular disease (CVD) or from non-CVD consequences of cardiovascular risk factors.

e YPLL rate refers to years of potential life lost per thousand adults due to these deaths.

f Risk management costs refers to per-adult costs of individual-level clinical and behavioral management of cardiovascular risk factors. Costs are expressed in 2008 dollars per adult and have been discounted back to 2012 using an annual discount rate of 3% per year.

g Acute/extended care costs refers to per-adult costs of acute, rehabilitation, and disability care resulting from cardiovascular events or from other disease attributable to cardiovascular risk factors. Costs are expressed in 2008 dollars per adult and have been discounted back to 2012 using an annual discount rate of 3% per year.

h Productivity costs refers to per-adult loss of paid or household work contribution due to deaths, disability, and hospitalization from cardiovascular events or attributable to cardiovascular risk factors. Costs are expressed in 2008 dollars per adult and have been discounted back to 2012 using an annual discount rate of 3% per year.

i Combined costs refers to the sum of risk management, acute/extended care, and productivity costs. Costs are expressed in 2008 dollars per adult and have been discounted back to 2012 using an annual discount rate of 3% per year.

The air and lifestyle interventions had modest effects through 2020. The combined air interventions led to modest reductions in the death rate (−2.3%; 95% uncertainty range, −2.6% to −1.9%) and combined costs (−1.9%; 95% uncertainty range, −2.2% to −1.6%). The health and cost effects were evenly divided between the established and emerging air interventions. The combined lifestyle interventions provided greater reductions in the death rate (−5.7%; 95% uncertainty range, −7.3% to −4.4%) and combined costs (−4.3%; 95% uncertainty range, −5.5% to −3.3%). The emerging lifestyle interventions contributed more than the established interventions did.

The combined care, air, and lifestyle interventions resulted in large reductions in the death rate (−32.6%; 95% uncertainty range, −34.7% to −30.6%) and a moderate reduction in combined costs (−4.5%; 95% uncertainty range, −7.4% to −1.7%). Both established and emerging interventions contributed to these reductions, but the established interventions contributed more than the emerging ones did with respect to health effects, while the emerging interventions contributed more than the established ones did with respect to total costs.

### Cumulative results through 2040

We calculated cumulative results from testing the 6 intervention clusters from 2012 to 2040 (Table 3). The combined care interventions resulted in large reductions in the death rate (−47.7%; 95% uncertainty range, −51.9% to −45.7%) and a moderate reduction in combined costs (−9.4%; 95% uncertainty range, −14.6% to −6.1%). Both established and emerging care interventions contributed to these improvements, but the established interventions contributed more than the emerging ones did.

**Table 3:** Grouped Intervention Impacts on Deaths and Costs 2012 Through 2040

Outcome	% Change from Base Case^a^ (95% Uncertainty Range)^b^			Base Case^a^
	Established	Emerging	Combined	
**Care interventions^c^**				
Death rate^d^	−35.6 (−40.6 to −31.0)	−17.2 (−18.2 to −16.3)	−47.7 (−51.9 to −45.7)	5.74
YPLL rate^e^	−34.8 (−39.3 to −30.5)	−15.5 (−16.5 to −14.6)	−45.9 (−49.7 to −44.1)	72.1
Risk management costs^f^	83.3 (76.5 to 89.9)	30.1 (23.8 to 38.2)	128.9 (114.0 to 143.3)	490
Acute and extended care costs^g^	−19.4 (−25.5 to −13.4)	−10.8 (−11.6 to −10.1)	−31.0 (−33.6 to −27.3)	539
Productivity costs^h^	−31.1 (−35.7 to −26.7)	−13.8 (−14.6 to −13.1)	−41.2 (−44.6 to −38.8)	1,769
Combined costs^i^	−8.8 (−13.5 to −4.6)	−5.5 (−6.9 to −4.0)	−9.4 (−14.6 to −6.1)	2,797
**Air interventions^c^**				
Death rate^d^	−3.5 (−4.2 to −2.8)	−2.3 (−2.8 to −1.9)	−5.6 (−6.3 to −4.8)	5.74
YPLL rate^e^	−3.2 (−3.8 to −2.5)	−2.0 (−2.5 to −1.7)	−5.0 (−5.6 to −4.2)	72.1
Risk management costs^f^	−1.7 (−2.0 to −1.2)	−0.5 (−0.6 to −0.4)	−2.1 (−2.5 to −1.7)	490
Acute and extended care costs^g^	−2.6 (−3.2 to −2.1)	−2.3 (−2.9 to −1.9)	−4.8 (−5.6 to −4.0)	539
Productivity costs^h^	−3.9 (−4.7 to −3.0)	−2.0 (−2.5 to −1.6)	−5.7 (−6.4 to −4.8)	1,769
Combined costs^i^	−3.3 (−4.0 to −2.6)	−1.8 (−2.2 to −1.5)	−4.9 (−5.6 to −4.1)	2,797
**Lifestyle interventions^c^**				
Death rate^d^	−1.5 (−2.4 to −1.7)	−9.7 (−11.8 to −7.6)	−10.8 (−12.8 to −8.5)	5.74
YPLL rate^e^	−1.5 (−2.4 to −1.7)	−8.6 (−10.4 to −6.7)	−9.8 (−11.5 to −7.7)	72.1
Risk management costs^f^	−1.0 (−1.6 to −0.4)	−7.1 (−8.1 to −5.8)	−7.9 (−8.9 to −6.5)	490
Acute and extended care costs^g^	−0.9 (−1.5 to −0.4)	−7.8 (−10.3 to −5.6)	−8.5 (−10.9 to −6.2)	539
Productivity costs^h^	−1.4 (−2.1 to −0.6)	−7.9 (−9.5 to −6.1)	−8.9 (−10.5 to −6.9)	1,769
Combined costs^i^	−1.2 (−1.9 to −0.5)	−7.7 (−9.4 to −6.0)	−8.6 (−10.2 to −6.7)	2,797
**All 3 intervention areas^c^**				
Death rate^d^	−37.7 (−42.4 to −33.2)	−25.0 (−26.7 to −23.1)	−51.3 (−54.1 to −48.7)	5.74
YPLL rate^e^	−36.8 (−41.1 to −32.6)	−22.4 (−23.9 to −20.7)	−49.2 (−51.7 to −47.0)	72.1
Risk management costs^f^	78.6 (71.6 to 85.7)	21.1 (14.5 to 28.6)	107.0 (94.0 to 122.7)	490
Acute and extended care costs^g^	−21.6 (−27.5 to −15.8)	−18.7 (−21.1 to −16.1)	−36.6 (−41.2 to −31.9)	539
Productivity costs^h^	−33.5 (−37.6 to −29.2)	−20.2 (−21.5 to −18.7)	−44.6 (−47.1 to −42.2)	1,769
Combined costs^i^	−11.5 (−16.1 to −7.3)	−12.7 (−14.4 to −10.6)	−16.5 (−20.7 to −12.8)	2,797

Abbreviation: YPLL, years of potential life lost.

a Base case results are cumulative averages from model simulations with only baseline conditions for 2012 through 2040 .

b Percentage changes from base case with uncertainty ranges are comparisons of intervention test results against base case for 200 different model Monte Carlo calibrations defined by random sampling of 89 uncertain effect-size parameters. The number not in parentheses is the median of the 200 comparisons; the 2 numbers in parentheses are the 2.5 percentile and 97.5 percentile of the 200 comparisons, respectively.

c Interventions ramp up to full strength over 5 years, from 2012 to 2017.

d Death rate refers to deaths per thousand adults from cardiovascular disease (CVD) or from non-CVD consequences of cardiovascular risk factors.

e YPLL rate refers to years of potential life lost per thousand adults due to these deaths.

f Risk management costs refers to per-adult costs of individual-level clinical and behavioral management of cardiovascular risk factors. Costs are expressed in 2008 dollars per adult and have been discounted back to 2012 using an annual discount rate of 3% per year.

g Acute/extended care costs refers to per-adult costs of acute, rehabilitation, and disability care resulting from cardiovascular events or from other disease attributable to cardiovascular risk factors. Costs are expressed in 2008 dollars per adult and have been discounted back to 2012 using an annual discount rate of 3% per year.

h Productivity costs refers to per-adult loss of paid or household work contribution due to deaths, disability, and hospitalization from cardiovascular events or attributable to cardiovascular risk factors. Costs are expressed in 2008 dollars per adult and have been discounted back to 2012 using an annual discount rate of 3% per year.

i Combined costs refers to the sum of risk management, acute/extended care, and productivity costs. Costs are expressed in 2008 dollars per adult and have been discounted back to 2012 using an annual discount rate of 3% per year.

The combined air interventions resulted in moderate reductions in the death rate (−5.6%; 95% uncertainty range, −6.3% to −4.8%) and combined costs (−4.9%; 95% uncertainty range, −5.6% to −4.1%). Both established and emerging air interventions contributed to these improvements, but the established air interventions contributed more than the emerging ones did.

The combined lifestyle interventions resulted in moderate reductions in the death rate  (−10.8%; 95% uncertainty range, −12.8% to −8.5%) and combined costs (−8.6%; 95% uncertainty range, −10.2% to −6.7%). Both established and emerging lifestyle interventions contributed to these improvements, but the emerging interventions contributed more than the established interventions did.

The combined care, air, and lifestyle interventions resulted in large reductions in the death rate (−51.3%; 95% uncertainty range, −54.1% to −48.7%) and a moderate reduction in combined costs (−16.5%; 95% uncertainty range, −20.7% to −12.8%). Both established and emerging interventions contributed to these improvements, but the established interventions contributed more than the emerging interventions did with respect to health impacts, whereas the emerging interventions contributed more than the established ones with respect to total costs.

### Sensitivity to uncertainties

Results from the probabilistic sensitivity analysis are represented in the uncertainty ranges (Table 2, Table 3). All of the results described above regarding the relative importance of established and emerging interventions within each of the 3 policy domain areas and in total are not qualitatively affected by uncertainties.

## Discussion

We used PRISM to compare the health and economic effects of established and emerging public health interventions in care, air, and lifestyle policy domains. Our testing suggests that established interventions may still have much to contribute to reducing deaths through greater use of well-known approaches to preventive and acute clinical care, whereas emerging interventions have the potential to contribute significantly through certain types of preventive care and improved nutrition (especially trans fat and sodium reduction). Considering all areas of intervention (care, air, and lifestyle) together, emerging interventions have the potential to improve health significantly, but not by as much as further expansions in established interventions. The emerging interventions have the potential to reduce costs significantly, perhaps even somewhat more than the established interventions do, because they avoid large increases in the costs of risk factor management.

The results reported here may be compared with those of other modeling studies, such as Kahn et al (16) and Kottke et al (17), that have assessed the potential impact of multiple CVD interventions individually and in combination. The most directly comparable study is Kahn et al, which used the Archimedes microsimulation model to analyze 11 nationally recommended prevention activities. These activities were included in our care risk factors, some of them established (eg, control of existing hypertension, high cholesterol, and diabetes; pre-CVD aspirin use, smoking cessation, and weight reduction in the obese) and one emerging, control of prediabetes. The Archimedes model projects that expanding all 11 activities by feasible amounts over the next 30 years could decrease myocardial infarctions by 36% and strokes by 20%. The greatest benefits are projected to come from pre-CVD aspirin use, prediabetes control, weight reduction, diabetes control, and post-CVD cholesterol control. By comparison, in PRISM, when all 34 of the care interventions were implemented to their best plausible levels, coronary heart disease events (including myocardial infarctions and episodes of angina pectoris) were reduced by 50% and strokes by 56% by the year 2040, relative to the base case (results available upon request). We have not attempted a head-to-head comparison of PRISM and Archimedes using equivalent assumptions, but even the imperfect comparison above is helpful. First, PRISM, with its broader array of preventive care interventions, suggests the potential for more reduction in CV events than Archimedes does, as expected. Second, the Archimedes study is consistent with our conclusion that established plus emerging interventions, if they can be implemented successfully, would provide significantly greater impact than established interventions alone.

Our results are subject to several limitations. First, the simplified compartmental structure of PRISM means that it cannot capture certain detailed comorbidity effects and distributional effects that one might see in a microsimulation like Archimedes. The advantage of the compartmental approach is that a simulation can be performed in an instant, and thorough sensitivity testing of dozens of assumptions (as we have done here) thereby becomes practical (19,20).

Second, although PRISM does model changes in the age distribution of the population, it does not model changes over time in other demographic characteristics, such as race/ethnicity, urban/rural residence, or education and income levels. Nonetheless, the significance of such characteristics can be explored by calibrating the model to represent localities that are demographically different from the United States overall. Such calibrations have been performed with PRISM and suggest that the general findings here are unaffected by differences in demographics (15,24).

Third, although the combined costs calculated in PRISM included indirect productivity costs and several types of direct medical costs, including the direct costs of the model’s 34 care interventions, they do not include the implementation and enforcement costs of the model’s 16 air and lifestyle interventions. One study suggests that comprehensive population-level prevention (preventing smoking, increasing physical activity, and improving nutrition) could be accomplished for $10 per person per year (26). This amount is 100 times smaller than the nearly $1,000 per person per year that PRISM calculates would be required in additional preventive care costs (ie, implementation costs for care levers) for the model’s 34 care interventions, which still manage to be cost-effective. Thus, the cost savings from the air and lifestyle interventions are likely to be much larger than their implementation costs, and our policy findings are likely insensitive to the presence or absence of these costs in the analysis.

Fourth, the comparisons in this study have used certain outcome measures for deaths and costs. These are arguably good measures but others, such as cost per quality-adjusted life year, might yield different results. Fifth, as with all models, the PRISM estimates of effect sizes and other parameters are based on data and studies available at the time of model creation. As research evolves, some parameter estimates may become outdated. We have regularly updated PRISM to reflect the latest data and studies; the work presented here reflects a model update performed in November 2013.

Despite these limitations, PRISM provides public health officials a unique analytic platform for examining the potential influence of different kinds of interventions, both established and emerging, for reducing CVD risks. Our modeling suggests that established interventions could still contribute much toward reducing deaths and costs through improvements in preventive and acute care, whereas emerging interventions have the potential to contribute significantly through other types of preventive care and improved nutrition.
